# Loss of TFF1 promotes *Helicobacter pylori*-induced β-catenin activation and gastric tumorigenesis

**DOI:** 10.18632/oncotarget.3772

**Published:** 2015-04-27

**Authors:** Mohammed Soutto, Judith Romero-Gallo, Uma Krishna, M. Blanca Piazuelo, M. Kay Washington, Abbes Belkhiri, Richard M. Peek, Wael El-Rifai

**Affiliations:** ^1^ Department of Veterans Affairs, Tennessee Valley Healthcare System, Nashville, Tennessee, USA; ^2^ Department of Surgery, Vanderbilt University Medical Center, Nashville, Tennessee, USA; ^3^ Division of Gastroenterology, Hepatology, & Nutrition, Vanderbilt University Medical Center, Nashville, Tennessee, USA; ^4^ Department of Pathology, Microbiology and Immunology, Vanderbilt University Medical Center, Nashville, Tennessee, USA; ^5^ Department of Cancer Biology, Vanderbilt University Medical Center, Nashville, Tennessee, USA

**Keywords:** TFF1, Helicobacter pylori, ß-catenin, gastric cancer

## Abstract

Using *in vitro* and *in vivo* models, we investigated the role of TFF1 in suppressing *H. pylori*-mediated activation of oncogenic β-catenin in gastric tumorigenesis. A reconstitution of TFF1 expression in gastric cancer cells decreased *H. pylori*-induced β-catenin nuclear translocation, as compared to control (*p* < 0.001). These cells exhibited significantly lower β-catenin transcriptional activity, measured by pTopFlash reporter, and induction of its target genes (*CCND1* and *c-MYC)*, as compared to control. Because of the role of AKT in regulating β-catenin, we performed Western blot analysis and demonstrated that TFF1 reconstitution abrogates *H. pylori*-induced p-AKT (Ser473), p-β-catenin (Ser552), c-MYC, and CCND1 protein levels. For *in vivo* validation, we utilized the *Tff1*-KO gastric neoplasm mouse model. Following infection with PMSS1 *H. pylori* strain, we detected an increase in the nuclear staining for β-catenin and Ki-67 with a significant induction in the levels of *Ccnd1* and *c-Myc* in the stomach of the *Tff1*-KO, as compared to *Tff1-*WT mice (*p* < 0.05). Only 10% of uninfected *Tff1*-KO mice, as opposed to one-third of *H. pylori-*infected *Tff1*-KO mice, developed invasive adenocarcinoma (*p* = 0.03). These findings suggest that loss of TFF1 could be a critical step in promoting the *H. pylori*-mediated oncogenic activation of β-catenin and gastric tumorigenesis.

## INTRODUCTION

Protection of the gastrointestinal tissue integrity is physiologically critical in the presence of persistent irritations caused by microbial flora and harmful agents. The repair of the gastric epithelial cells layer is mediated by several factors that comprise the trefoil factor protein 1 (TFF1) [1]. TFF1 interacts with mucin proteins to stabilize the mucus gel layers of the gastric epithelium and plays a dynamic role in the protection and regeneration of the gastric mucosa [2]. Several reports have demonstrated that *TFF1* functions as a gastric tumor suppressor gene [3, 4], and loss of *Tff1* leads to development of dysplastic lesions and adenocarcinoma in antropyloric gastric tissues in mice [5–7]. In human, *TFF1* expression is downregulated in approximately two-thirds of gastric adenocarcinoma due to deletion, loss of heterozygosity, hypermethylation, or transcription regulation [8–12]. A large study of approximately 300 gastric cancers has shown that TFF1 expression is significantly more reduced in cases with differentiated type and intestinal type gastric cancer [12]. This study has shown that a combination of reduced TFF1 expression and high TFF3 expression (*p* = 0.018) was determined as an independent prognostic factor significantly associated with poor OS in patients with early stage gastric cancer [12].

β-catenin signaling pathway has a well-established role in the regulation of cell growth and proliferation, and its constitutive oncogenic activation is frequent in human cancers, including gastric cancer [13, 14]. Several reports have shown that β-catenin activation occurs through its translocation to the nucleus; this process results in its binding to members of the TCF/LEF family of transcription factors [15]. The TCF/β-catenin complex activates target genes such as *c-MYC* and cyclin D1 (*CCND1*). Inhibition of β-catenin results in a decreased expression of its target genes and suppression of cell proliferation [15–17].

*Helicobacter pylori*, a gram-negative bacterium, was first discovered in 1983 by Marshall BJ and Warren JR [18]. The World Health Organization concluded that *H. pylori* is a definite carcinogen based on epidemiological evidence, and it is present in the stomach of approximately half of the world's population [19, 20]. *H. pylori* infection induces a sequence of events that begins with superficial gastritis, progressing towards chronic atrophic gastritis, intestinal metaplasia, dysplasia, and finally gastric cancer [21]. The cag pathogenicity island (cag-PAI) is the most characterized *H. pylori* virulence factor. CagA (cytotoxin-associated gene) is one of the 30 genes encoded by cag-PAI [22]. Several reports have investigated how CagA controls cellular effects through regulation of specific signal transduction pathways [23]. Among the intracellular signaling affected by CagA is β-catenin pathway [24–27].

In this study, we investigated the role of TFF1 in suppressing *H. pylori*-induced activation of β-catenin using *in vitro* and *in vivo* gastric neoplasm models. Our results suggest that TFF1 plays a critical protective role against *H. pylori*-mediated activation of oncogenic β-catenin where loss of TFF1 could be an important turning point in gastric tumorigenesis.

## RESULTS

### Reconstitution of TFF1 decreases *H. pylori*-induced nuclear localization and transcriptional activation of β-catenin

**Figure 1:**
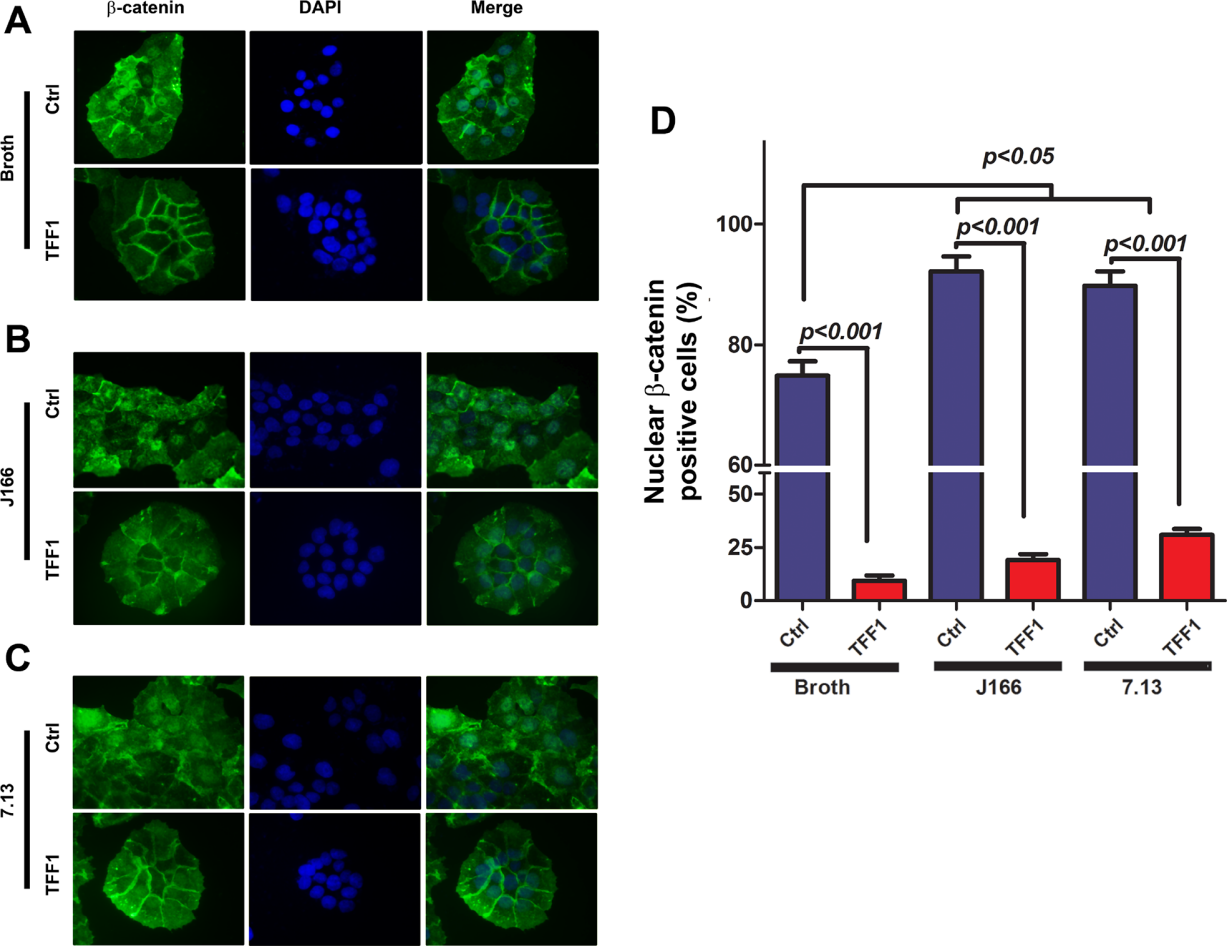
Reconstitution of TFF1 suppresses *H. pylori*-mediated nuclear localization of β-catenin **A–C.** Immunofluorescence assay of β-catenin in control and TFF1-expressing MKN28 cells with and without infection with *H. pylori*. (A) Uninfected cells. (B) Cells infected with *H. pylori* strain J166. (C) Cell infected with *H. pylori* strain 7.13. Nuclear localization of β-catenin is depicted in green. DAPI (blue) was used as a nuclear counterstain. Original magnification, × 400. **D.** Graph indicating the percentage of at least 200 counted cells that show β-catenin nuclear staining.

**Figure 2:**
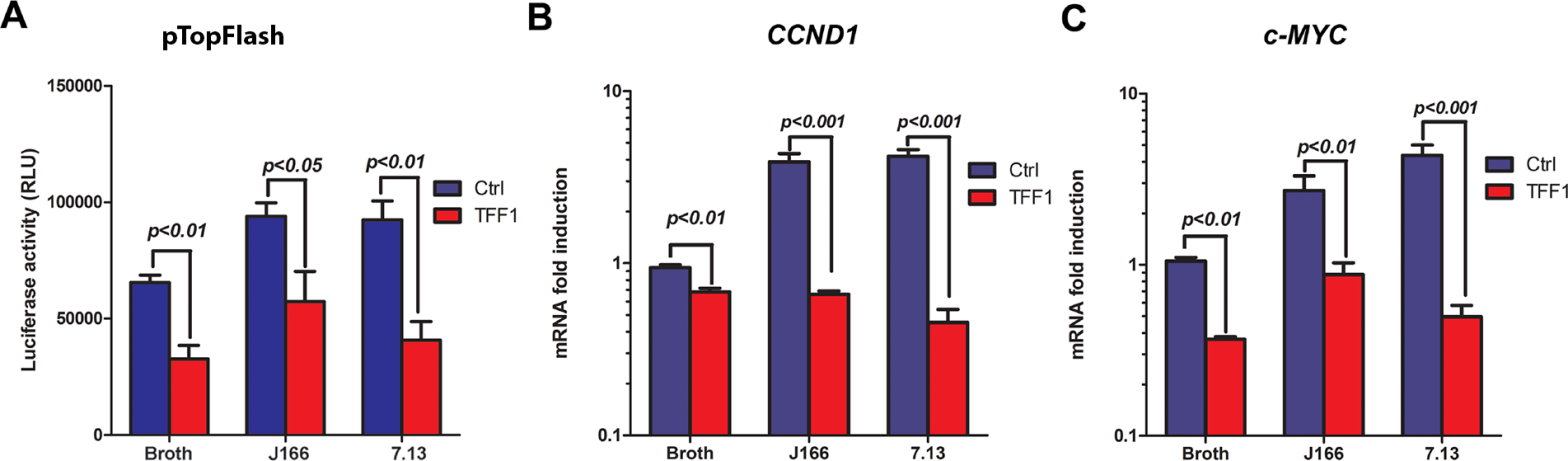
TFF1 abrogates *H. pylori*-induced transcriptional activation of β-catenin and its target genes mRNA expression **A.** pTopFlash luciferase reporter assays showing β-catenin and TCF-LEF transcriptional activity. TFF1 expressing cells showed a decrease of pTopFlash activity as compared to control cells after *H. pylori* infection. **B–C.** qRT-PCR data indicate mRNA downregulation of β-catenin target genes, *CCND1* (B) and *c-MYC* (C) in MKN28 cells expressing TFF1 as compared to control cells after infection with *H. pylori* J166 or 7.13 strains. These data are representative of at least three independent experiments.

In order to determine whether TFF1 reconstitution affects *H. pylori*-mediated activation of β-catenin, we performed immunofluorescence analysis and utilized two CagA+ *H. pylori* carcinogenic strains, J166 and 7.13. Using an adenovirus expression system, we transiently expressed TFF1 or control in MKN28 cells. After 48 h, the cells were infected with *H. pylori* J166 or 7.13 strains for 3 h. In MKN28 control cells, *H. pylori* infection induced a significant increase in β-catenin nuclear staining as compared to uninfected cells (Figure 1A–1D). However, the reconstitution of TFF1 expression significantly abrogated *H. pylori-*induced β-catenin nuclear translocation as compared to control cells (*p* < 0.001, Figure 1B–1D). In uninfected MKN28 control cells with β-catenin constitutively activated and localized in the nucleus, the reconstitution of TFF1 expression in these cells led to a nuclear decrease and a membrane increase of β-catenin staining (Figure 1A–1D). To investigate the suppressive effect of TFF1 on *H. pylori*-mediated β-catenin activation, we examined the TCF/LEF transcriptional activation using pTopFlash luciferase reporter. In *H. pylori* infected cells, TFF1 reconstitution significantly reduced the pTopFlash reporter activity as compared to the controls in J166 (*p* < 0.05) and 7.13 (*p* < 0.01) strains (Figure 2A). However, in control cells, infection with *H. pylori* without TFF1 expression induced a significant increase of pTopFlash reporter activity as compared to uninfected cells (Figure 2A, *p* < 0.01). Together, the immunofluorescence and reporter assays data clearly indicated that TFF1 expression decreases *H. pylori-*mediated β-catenin nuclear localization and transcriptional activation in gastric cancer cells.

### TFF1 abrogates *H. pylori*-induced mRNA and protein expression of β-catenin target genes

**Figure 3:**
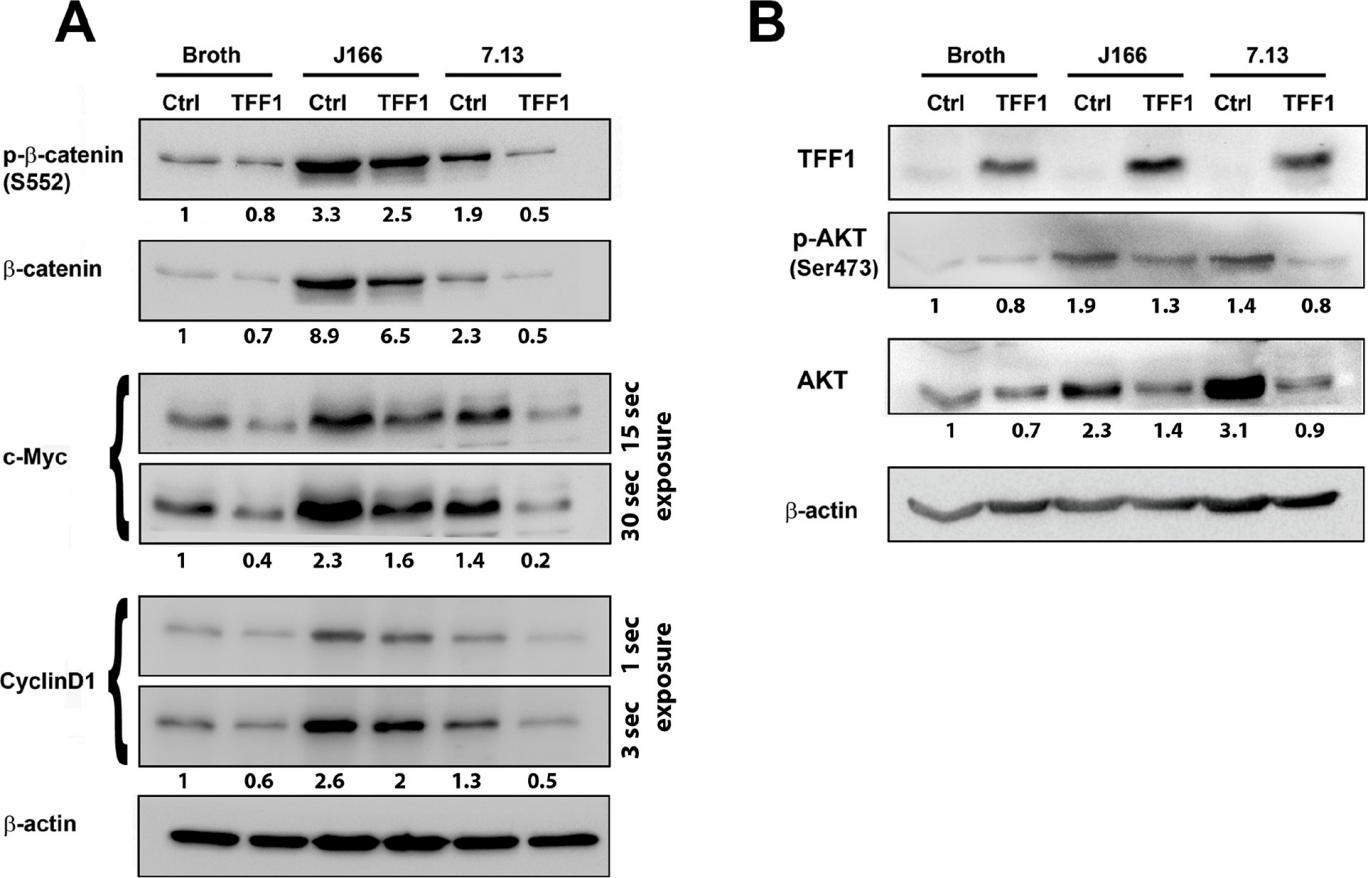
Reconstitution of TFF1 suppresses protein expression of β-catenin target genes in *H. pylori*-infected cells **A–B.** Western blot analysis of β-catenin downstream and upstream target genes. Reconstitution of TFF1 expression in *H. pylori-*infected MKN28 cells led to a decrease in protein levels of phospho-β-catenin (S552), β-catenin, c-MYC, and Cyclin D1 (A); phospho-AKT (Ser473) and AKT (B), as compared to their respective *H. pylori*-infected control cells. Protein loading was normalized for equal levels of β-actin. The intensity ratios of the indicated proteins level relative to Broth control were calculated after normalizing to β-actin using the ImageJ software (NIH). The Western blot results are representative of three independent experiments.

To investigate whether TFF1-dependent suppression of *H. pylori*-induced transcriptional activation of β-catenin leads to downregulation of β-catenin downstream targets, we assessed mRNA and protein expression of cyclin D1 and c-Myc in an *in vitro* MKN28 cancer cell model. After infection with *H. pylori* strains, qRT-PCR results indicated a significant decrease of mRNA expression of *CCND1* (Figure 2B, *p* < 0.001 for both J166 and 7.13 strains) and *c-MYC* (Figure 2C, *p* < 0.01 and *p* < 0.001 for J166 and 7.13 strains, respectively) in TFF1 expressing cells as compared to control cells. In addition, *H. pylori* infection of control cells significantly increased *CCND1* and *c-MYC* mRNA levels as compared to uninfected cells (Figure 2B–2C). In accordance with mRNA results and after infection with *H. pylori* strains, J166 and 7.13, Western blot analysis demonstrated a decrease of phospho-β-catenin (S552), β-catenin, c-Myc and cyclin D1 protein levels in MKN28 cells expressing TFF1 as compared to control cells (Figure 3A). We next investigated whether the reconstitution of TFF1 expression abrogates *H. pylori*-induced activation of AKT, a major regulator of β-catenin pathway. Our data indicated that after infection with both strains of *H. pylori* (J166 and 7.13), phospho-AKT (Ser473) and AKT protein levels were decreased in TFF1 expressing cells as compared to control cells (Figure 3B). Furthermore, the data showed that *H. pylori* infection of control cells increased expression of all analyzed proteins as compared to uninfected cells (Figure 3A–3B). Collectively, the data demonstrated that TFF1 negatively regulates *H. pylori*-induced β-catenin activation of target genes *in vitro*.

### Loss of Tff1 promotes *H. pylori*-mediated activation of β-catenin and nuclear localization in gastric neoplasm mouse model

**Figure 4:**
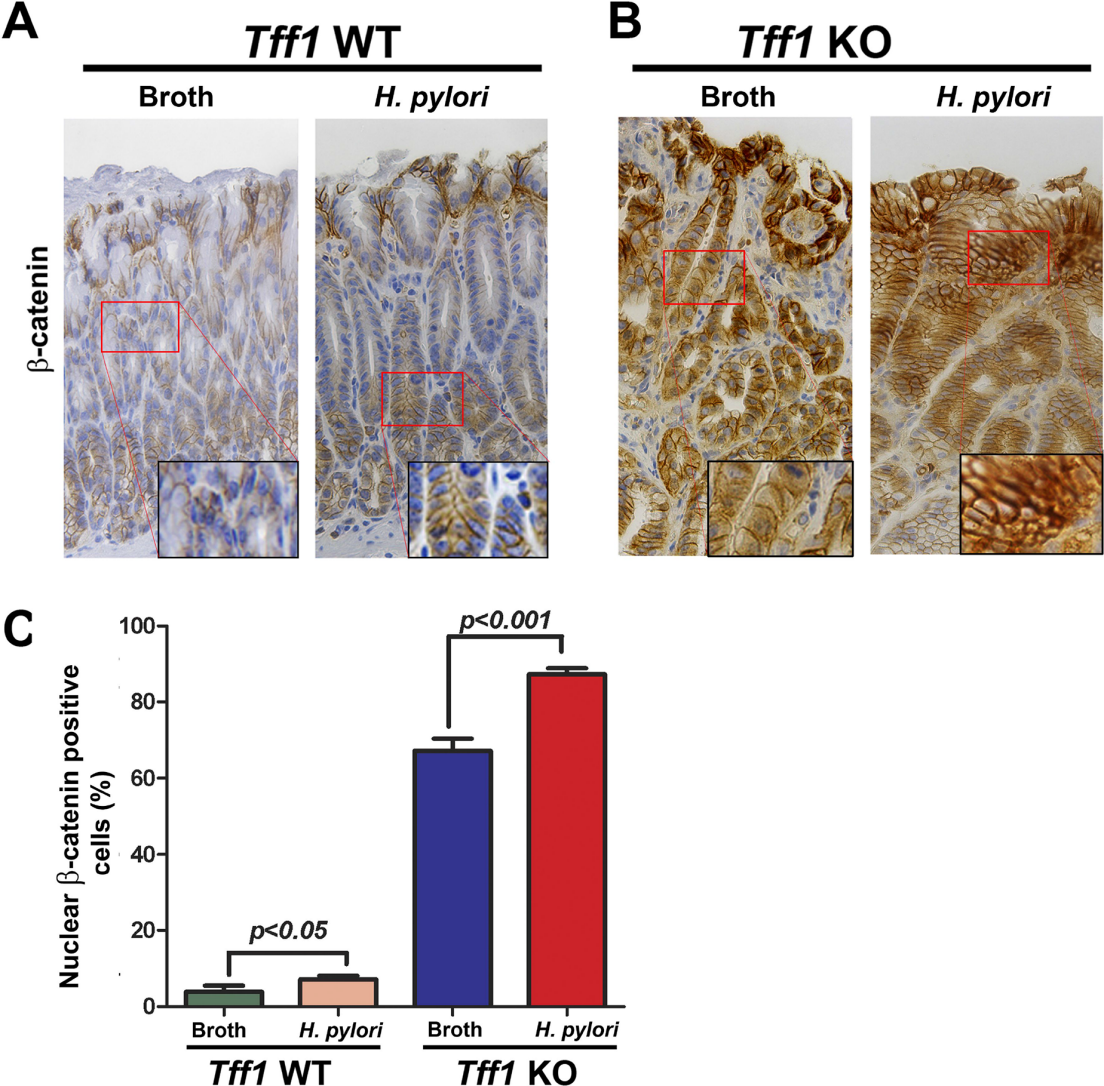
*H. pylori* infection enhances nuclear accumulation of β-catenin in *Tff1* knockout gastric cancer mouse model **A–B.** Immunohistochemistry analysis of β-catenin expression in the antropyloric gastric mucosa from uninfected or *H. pylori* (PMSS1)-infected *Tff1*-wild-type (*Tff1-*WT) mice (9–10 months of age) (A) and *Tff1*-knockout (*Tff1-*KO) mice (9–10 months of age) (B), mice were challenged at 6–8 weeks of age with either sterile *Brucella* broth or *H. pylori* strain (PMSS1) by oral gavage. **C.** Quantification of nuclear β-catenin-positive staining in at least 200 counted cells presented as percentage ± SEM is shown.

**Figure 5:**
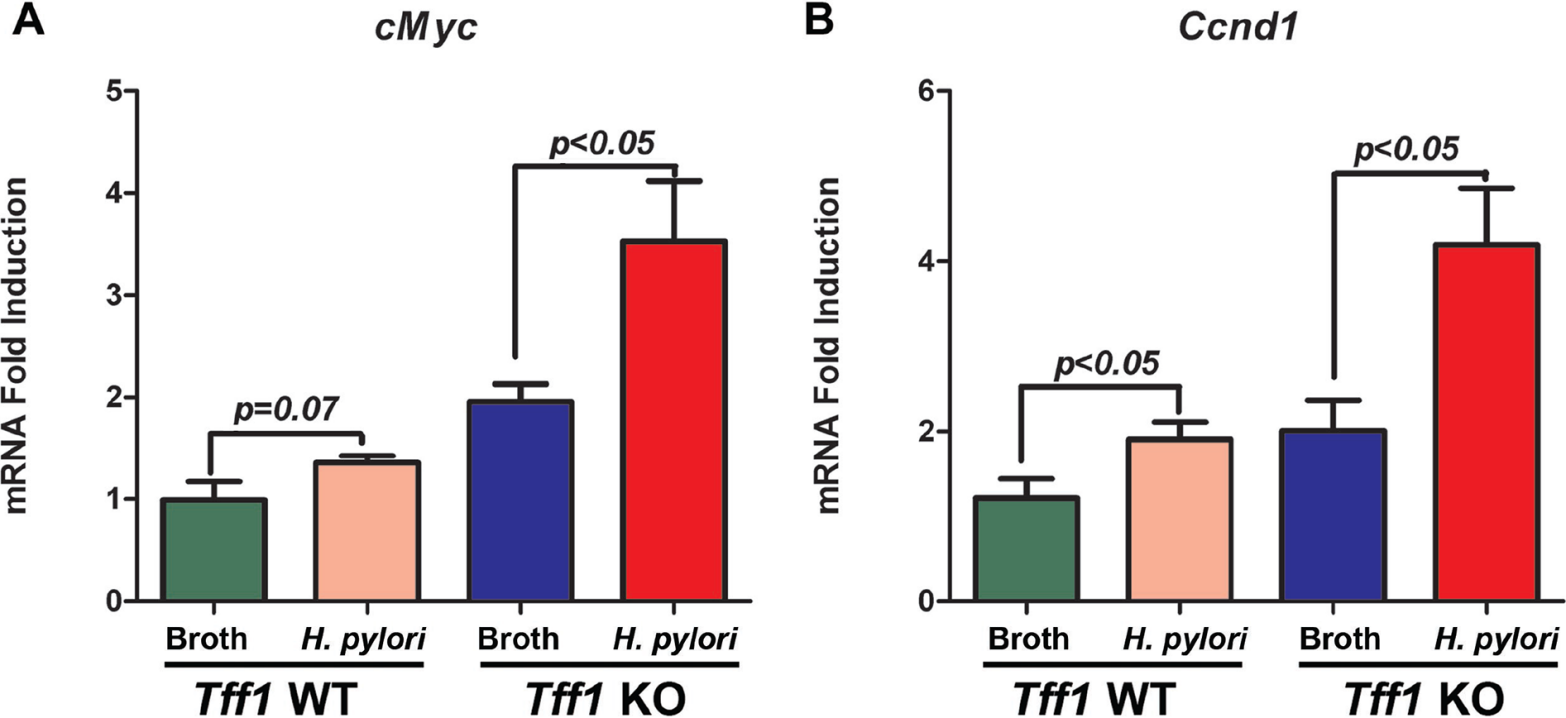
*H. pylori* infection increases mRNA expression of β-catenin target genes in *Tff1*-knockout mice gastric tissues **A–B.** Quantitative RT-PCR analysis showing mRNA expression levels of β-catenin target genes, *c-Myc* (A) and *Ccnd1* (B) in uninfected or *H. pylori* strain (PMSS1) infected *Tff1-*WT and *Tff1*-KO mice (12 weeks of age, 10 mice each).

To extend our *in vitro* investigation into an *in vivo* gastric neoplasm mouse model, *Tff1*-KO and *Tff1*-WT mice (6–8 weeks of age) were challenged with Brucella broth as uninfected controls or with *H. pylori* PMSS1 *cagA+* strain. Immunohistochemistry analysis of the antropyloric region of the gastric tissue from *Tff1*-WT-infected mice showed a small but significant (*p* < 0.05) increase of nuclear β-catenin staining as compared to uninfected *Tff1*-WT mice (Figure 4A). However, in infected *Tff1-KO* mice, the immunostaining showed a significant (*p* < 0.001) increase of β-catenin nuclear localization as compared to uninfected *Tff1*-KO mice (Figure 4B). The quantification of the immunohistochemistry data demonstrated a significant increase of the percentage of β-catenin nuclear staining in uninfected *Tff1*-KO mice as compared to uninfected *Tff1*-WT mice (Figure 4C). However, in *H. pylori*-infected *Tff1*-KO mice, our analysis showed an even more significant (*p* < 0.001) increase of β-catenin nuclear staining as compared to uninfected *Tff1*-KO (Figure 4C). Next, we examined the mRNA expression of β-catenin downstream target genes in gastric epithelial cells from infected and uninfected mice. The qRT-PCR data confirmed the immunohistochemistry results and indicated a significant increase of *c-Myc* and *Ccnd1* mRNA expression in *Tff1*-KO as compared to *Tff1*-WT mice following *H. pylori* infection (Figure 5A–5B). The data also showed that *H. pylori* infection significantly increased *c-Myc* and *Ccnd1* mRNA levels in *Tff1*-KO mice as compared to uninfected mice (Figure 5A–5B). Collectively, these data clearly indicate that loss of *Tff1* in addition to *H. pylori* infection increase the activation and nuclear localization of β-catenin in our *Tff1-KO* gastric cancer mouse model.

### *H. pylori* infection augments gastric cell proliferation and tumorigenesis in *Tff1*-KO mice

**Figure 6:**
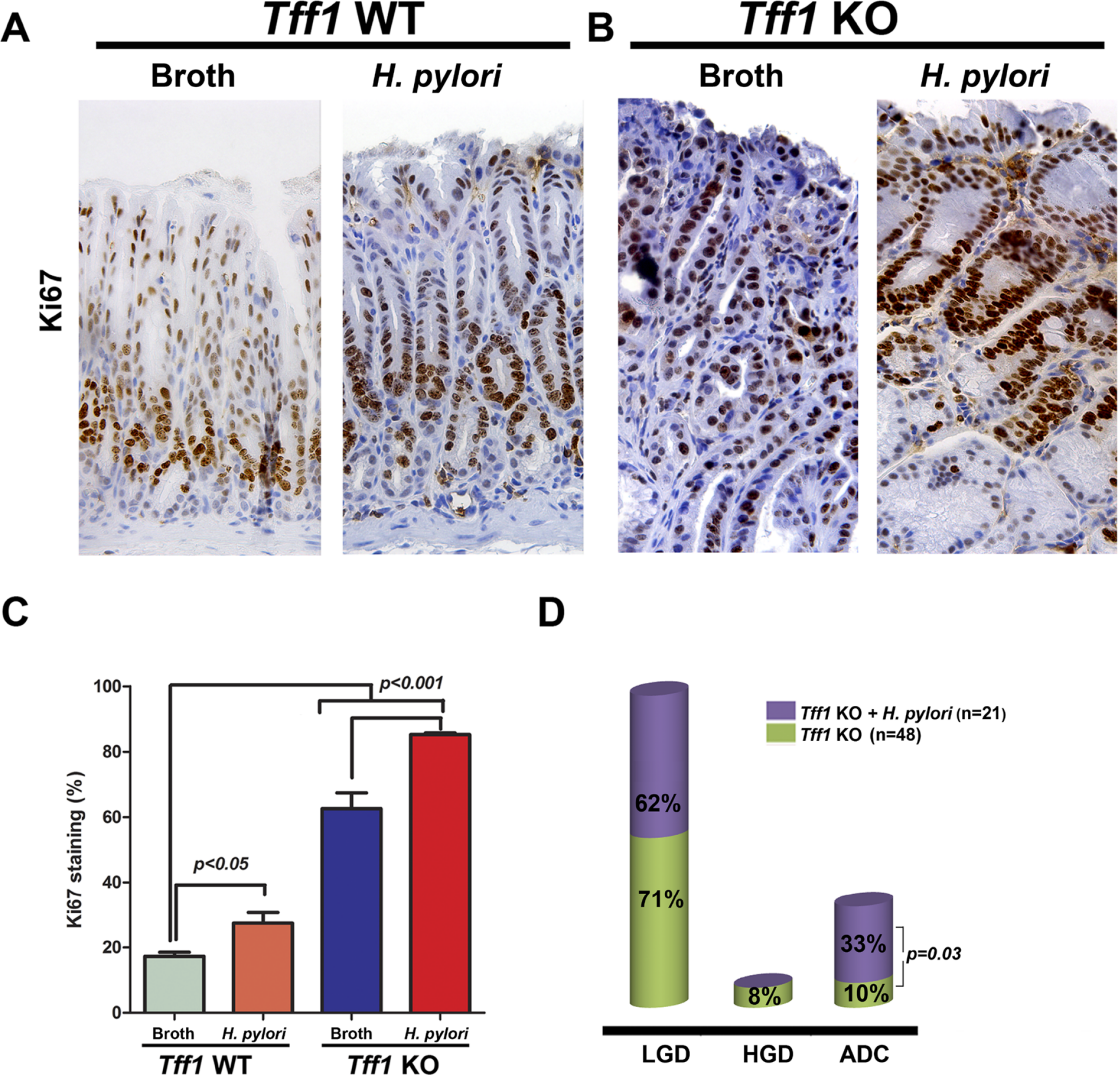
*H. pylori* infection enhances gastric cell proliferation and tumorigenesis in *Tff1*-knockout mice **A–B.** Representative images of Ki-67 immunostaining (brown nuclei) of serial sections, as in Figure 3, of gastric glands in *Tff1-*WT (9–10 months of age) (A) and *Tff1-*KO (9–10 Months of age) (B) uninfected or *H. pylori-*infected mice. Original magnification (× 100) was shown. **C.** The quantitative data of Ki-67 immunostaining in at least 200 counted cells were presented as percentage ±SEM. **D.** A diagram showing the percentage of uninfected or infected *Tff1-*KO mice with low-grade dysplasia (LGD), high-grade dysplasia (HGD), or invasive adenocarcinoma (ADC).

Next, we investigated whether *H. pylori* infection in combination with Tff1 loss could further increase cell proliferation, a hallmark function of β-catenin activation, and would subsequently increase the rate of tumorigenesis. We subjected gastric tissues from *Tff1*-WT and *Tff1*-KO uninfected or *H. pylori* (PMSS1 strain)-infected mice similar in age to immunohistochemical staining of Ki-67. In the *H. pylori*-infected *Tff1*-WT mice, Ki-67 staining was observed in the basal proliferative region of the gastric glands similar to the uninfected *Tff1*-WT mice with a slight increase of the percentage of Ki-67 positive nuclear staining (Figure 6A–6C, *p* < 0.05). However, the gastric tissues in the infected *Tff1*-KO mice exhibited a significant increase (*p* < 0.001) of the percentage of positive nuclear Ki-67 staining as compared to uninfected *Tff1*-KO mice. Of note, the Ki-67 staining extended to the surface of the mucosa in both infected and uninfected *Tff1*-KO mice (Figure 6B–6C).

Because the proliferation-associated marker Ki-67 is a characteristic of the antropyloric gastric lesions, we investigated the histological changes in uninfected and infected mice with *H. pylori*. Histological analysis using H&E staining indicated a marked increase of invasive adenocarcinoma occurrence among infected *Tff1*-KO mice (7 out of 21, 33%) as compared to age-matched (8–15 month old mice) uninfected *Tff1*-KO mice (5 out of 48, 10%), using our inventory from 2009–2014 (Figure 6D). However, both infected and uninfected *Tff1*-KO mice showed no significant difference in occurrence of LGD (62% and 71% respectively) (Figure 6D). Conversely, *Tff1*-WT mice infected with *H. pylori* did not develop any dysplastic or invasive gastric lesions. Taken together, the data demonstrated that TFF1 is a negative regulator of *H. pylori*-induced β-catenin activation *in vivo* and *in vitro*. The loss of TFF1 promotes the progression of histological lesions in response to *H. pylori* infection.

## DISCUSSION

Despite accumulating evidence from *in vitro* studies on the role of *H. pylori* in activating several oncogenic pathways, such as β-catenin [28–30], there is less information about the contribution of molecular events to *H. pylori-mediated* gastric tumorigenesis. Several studies have reported that long-term infection with *H. pylori* alone can induce chronic gastritis, chronic atrophic gastritis, and hyperplastic gastritis but is not sufficient to induce gastric cancer in wild-type mice [24, 31, 32]. We and others have demonstrated that silencing of *TFF1* promotes gastric tumorigenesis and cancer development in mouse and human [4, 5, 7, 33]. In this report, we investigated the role of the TFF1 in regulating *H. pylori-*mediated activation of β-catenin and gastric tumorigenesis. We provided evidence that TFF1 suppresses *H*. *pylori*-induced β-catenin activation *in vitro* and *in vivo*.

*H. pylori* plays an important role in activation of β-catenin through its oncoprotein CagA; this activation is dependent on its translocation and nuclear accumulation [34–36]. In accordance with the reported data, our *in vitro* results demonstrated an increase of β-catenin activation, as indicated by its nuclear accumulation and the pTopFlash reporter data after *H. pylori* infection. In contrast, the reconstitution of TFF1 expression reduced *H. pylori*-induced β-catenin activation. The *in vivo* data showed that *H. pylori* infection of *Tff1*-WT mice induced a slight increase of β-catenin nuclear localization, indicating its activation in accordance with previously reported findings [34, 35]. However, in *H. pylori-*infected *Tff1*-KO mice, β-catenin was highly activated as indicated by the increased percentage of nuclear β-catenin positive gastric epithelial cells. While we have previously shown that TFF1 negatively regulates β-catenin [7], our present findings suggest that TFF1 plays an important role in overcoming and suppressing *H. pylori*-mediated activation of oncogenic β-catenin, where loss of Tff1 accelerated the progression towards invasive gastric adenocarcinoma in mice.

Increased activation and mutation of the β-catenin gene have been demonstrated in various cancers [37]. Notably, approximately 26% of gastric carcinomas have been reported to carry β-catenin activating mutations [38]. The expression of β-catenin target genes *c-MYC* and cyclin D1 (*CCND1*), which promote tumorigenesis, is increased within *H. pylori* colonized human gastric mucosa and during coculture of *H. pylori* with gastric epithelial cells *in vitro* [26, 39, 40]. Our data demonstrated that the reconstitution of TFF1 expression in gastric cancer cells abrogated *H. pylori*-induced activation of β-catenin and decreased mRNA and protein expression of c-myc and cyclin D1. Conversely, loss of Tff1 expression in *H. pylori-*infected mice significantly increased *c-Myc* and *Ccnd1* mRNA expression; this increase was significantly enhanced as compared to uninfected *Tff1*-KO mice. This confirms the suppressive effect of TFF1 on *H. pylori*-induced activation of β-catenin *in vivo* and *in vitro*. These results suggest that *H. pylori* capitalizes on TFF1 loss to increase activation of β-catenin transcription activity and expression of oncogenes.

Colonization of gastric mucosa by *H. pylori* and deregulation of β-catenin signaling have been reported to induce epithelial hyperproliferation, which increase the risk for gastric adenocarcinoma [26, 41]. Our data demonstrated that *H. pylori* infection significantly enhances cell proliferation and the incidence of invasive gastric adenocarcinoma in the *Tff1*-KO mice. Interestingly, our results suggest that Tff1 plays a protective role in the stomach by suppressing *H. pylori*-induced activation of β-catenin. Based on our results, infection of *Tff1*-WT mice with *H. pylori* alone was not sufficient to drive gastric tumorigenesis, a finding that is consistent with epidemiological studies that demonstrate that almost half of the world's population is infected with *H. pylori* with less than 1% progressing to gastric cancer [42, 43]. These data suggest that additional molecular hits are required to promote *H. pylori*-mediated gastric tumorigenesis; loss of Tff1 is, therefore, plausibly a high-risk molecular event. Of note, approximately two-thirds of human gastric cancers, where *H. pylori* is a risk factor, demonstrate downregulation of TFF1 expression [9, 11, 12]. Our *in vivo* study demonstrated a significantly increased incidence of invasive adenocarcinoma in *H. pylori* infected *Tff1*-KO mice as compared to uninfected *Tff1*-KO mice. However, only one-third of the *H. pylori* infected *Tff1*-KO mice developed invasive gastric adenocarcinoma. This finding may suggest that multiple *H. pylori* infections and/or a longer follow-up are needed to obtain a higher incidence of invasive gastric adenocarcinoma in the *Tff1*-KO mouse model.

In summary, our study demonstrates that TFF1 plays an essential protective role against *H. pylori*-mediated oncogenic activation of β-catenin. The loss of TFF1 could define a turning point towards activation of oncogenic β-catenin and expression of oncogenes in gastric tumorigenesis.

## MATERIALS & METHODS

### Ethics statement

Procedures and experiments with mice were authorized by the Animal Care and Use Committee of the University of Vanderbilt and were carried out according to the guidelines.

### Cell culture and reagents

Human gastric cancer MKN28 cells were obtained from ATCC (Manassas, VA). These cells were maintained in Dulbecco's modified Eagle's medium (DMEM; GIBCO, Carlsbad, CA) supplemented with 10% fetal bovine serum (FBS; Invitrogen, Carlsbad, CA) and 1% penicillin/streptomycin (GIBCO). Cells were maintained at 37°C in an atmosphere containing 5% CO2. Specific antibodies against phospho-AKT (Ser473), AKT, phospho-β-catenin (Ser552), β-catenin, Cyclin D1 and β-actin were purchased from Cell Signaling Technology (Beverly, MA). c-Myc antibody was obtained from Santa Cruz Biotechnology, Inc. (Santa Cruz, CA).

### Reconstitution of TFF1 expression

To reconstitute the expression of TFF1 in MKN28 cells, we used an adenovirus expression system. We sub-cloned the *TFF1* coding sequence from the pcDNA3.1/TFF1 plasmid into the adenoviral shuttle vector (pACCMV). The recombinant adenovirus-expressing TFF1 was generated by co-transfecting HEK-293 cells with the shuttle and backbone adenoviral (pJM17) plasmids using the Calcium Phosphate Transfection kit (Applied Biological Materials, Richmond, BC). After infection, the MKN28 cells were analyzed for the expression of *TFF1* by qRT-PCR.

### *H. pylori* bacterial strains and culture conditions

For the *in vitro* studies, we used two CagA+ *H. pylori* carcinogenic strains, “J166” a clinical isolate of human-derived *H. pylori*, and “7.13” a rodent adapted strain derived from B128 *H. pylori*, which have been described previously [24, 44–47]. In the *in vivo* study, we used the wild-type rodent-adapted *cagA+ H. pylori* strain (PMSS1), a clinical isolate from a duodenal ulcer patient and the parental strain of the mouse-derivative Sydney strain 1 (SS1) [48]. All *H. pylori* plate cultures were performed on Brucella agar (BBL/Becton Dickinson, Sparks, MD) supplemented with 5% heat-inactivated newborn calf serum (Invitrogen) and ABPNV (amphotericin B, 20 mg/liter; bacitracin, 200 mg/liter; polymyxin B, 3.3 mg/liter; nalidixic acid, 10.7 mg/liter; vancomycin, 100 mg/liter) antibiotics (all from Sigma-Aldrich, Milwaukee, WI). *H. pylori* MPSS1 strain liquid cultures for mouse inoculation were grown in Brucella broth (BD Biosciences) with 5% NCS and antibiotic supplementation for approximately 24 h, pelleted by centrifugation, and suspended in Brucella broth.

### Immunofluorescence assay

MKN28 cells infected with control or TFF1 adenoviruses (10 MOI) were plated in 8-well chambers. After 48 h, cells were infected with either *H. pylori* J166 or 7.13 strains (100:1) for 3 h. Cells were washed with PBS and fixed with fresh 4% paraformaldehyde solution for 15 min at room temperature. Cells were then washed twice with PBS, followed by incubation in 10% normal goat serum blocking solution (Zymed Laboratories, San Francisco, CA) for 20 min at room temperature in a humidified chamber. Cells were incubated in the specific primary antibody against β-catenin diluted in PBS (1:400) for 2 h at room temperature in a humidified chamber. Cells were washed 3 times in PBS and incubated in fluorescein isothiocyanate (FITC)-tagged secondary antibody (1:1, 000; Jackson Immunoresearch, West Grove, PA) for 45 min at room temperature in a humidified chamber. The cells were then washed in PBS, mounted with Vectashield/DAPI (Vector Laboratories, Burlingame, CA), and visualized using an Olympus BX51 fluorescence microscope (Olympus Co. Center Valley, PA). For quantification, ImageJ software (US National Institutes of Health) was used. The images were transformed into 8-bit and a region of interest (ROI) was randomly selected in the nucleus and cytoplasm. The ratio of integrated density in the nucleus versus cytoplasm was determined by measuring the intensity density of the ROI in the nucleus and cytoplasm. The percentage of cells that show β-catenin nuclear staining was determined based on the value of the density ratio; *a* value equal to or less than 1 was considered negative, *a* value more than 1 was considered positive.

### Luciferase reporter assay

To evaluate the transcriptional activity of TCF/β-catenin, we used the luciferase reporter construct, pTopFlash, with six TCF binding sites (Upstate Biotechnology, Waltham, MA). Fugene 6 was used for transfection as directed by the manufacturer's protocol (Roche Applied Science, Indianapolis, IN). After infection with TFF1 adenoviral particles (10 MOI) or control adenoviruses for 3 h, cells were transfected with pTopFlash. 48 h later, cells were infected with *H. pylori* strains J166 or 7.13 (100:1), and luciferase and β-galactosidase activity were measured as described previously [49]. The firefly luciferase activity was normalized to β-galactosidase activity and expressed as relative luciferase unit with ± standard error of the mean (SEM).

### Quantitative real-time RT-PCR

Total RNA was isolated using the RNeasy Mini kit (Qiagen, Germantown, MD) and cDNA synthesis was performed using an iScript cDNA Synthesis Kit (Bio-Rad, Hercules, CA). Primers specific for mouse and human genes were designed using the online software Primer 3 (http://frodo.wi.mit.edu/primer3/). The forward and reverse primers were designed to span two different exons for each gene (human: *c-MYC* and *CCND1;* mouse: *c-Myc and Ccnd1*), as previously described [7]. All primers were purchased from Integrated DNA Technologies (IDT, Coralville, IA). The qRT-PCR was performed using an iCycler (Bio-Rad), with the threshold cycle number determined by use of iCycler software version 3.0. Reactions were performed in triplicate and the threshold cycle numbers were averaged. The results of the genes were normalized to housekeeping genes, 18S for human and actin for mouse. Expression ratios were calculated according to the formula 2^(Rt–Et)^/2^(Rn–En)^ [50] where Rt is the threshold cycle number for the reference gene observed in the test samples; Et is the threshold cycle number for the experimental gene observed in the test samples, Rn is the threshold cycle number for the reference gene observed in the reference samples, and En is the threshold cycle for the experimental gene observed in the reference samples. Rn and En values were calculated as an average of all reference samples.

### Western blotting

Cell lysates were prepared in RIPA buffer containing Halt Protease and Phosphatase Inhibitor Cocktail (Pierce Biotechnology, Inc., Rockford, IL) and were centrifuged at 4,390 g for 10 min at 4°C. Protein concentration was measured using a Bio-Rad Protein Assay (Bio-Rad). Equal amounts of proteins (10–15 μg) from each sample were subjected to SDS/PAGE and transferred onto nitrocellulose membranes. Target proteins were detected by using specific antibodies.

### Animals infection

*Tff1*-KO mouse model of gastric tumorigenesis [3] and normal *Tff1*-WT mice, at 6–8 weeks of age, were inoculated by oral gavage with 1×10^9^ colony-forming units of *H. pylori* MPSS1 strain in 0.1 ml of Brucella broth or Brucella broth alone as a vehicle control [51]. Mice were euthanized at 4, 24, 32, and 48 weeks post-challenge (8–10 mice per group). The 4-week challenged mice, with non-dysplastic lesions, were used for qRT-PCR to detect early changes in the expression of β-catenin target genes; older mice groups were used for histological evaluation of progression. Frozen and formalin fixed paraffin-embedded stomach tissue samples were collected from *H. pylori*-infected and uninfected *Tff1*-KO and *Tff1*-WT mice. All procedures were approved by the Animal Care Committee of Vanderbilt University.

### Histologic evaluation and immunohistochemical assessment

Histopathological classification and grading of the gastric tissues were performed by our pathologists (MBP and MKW) on H&E stained sections. Immunohistochemistry analysis for β-catenin was performed on paraffin-embedded stomach tissues using β-catenin mouse monoclonal antibody (Cell Signaling). Serial sections of the same embedded tissues were used to detect proliferating cells with a mouse monoclonal antibody directed against the Ki-67 antigen (Sigma-Aldrich).

### Statistical analysis

Using GraphPad Prism software, a One-way ANOVA Newman-Keuls Multiple Comparisons Test was used to compare the differences between three or more groups, and a two-tailed Student's test was used to compare the statistical difference between two groups. The differences were considered statistically significant when the *P* value was < 0.05.

## Abbreviations

TFF1: trefoil factor 1
H. pylori: Helicobacter pylori
KO: knockout
TCF/LEF: T cell factor/lymphoid
